# Diversification of the cullin family

**DOI:** 10.1186/1471-2148-9-267

**Published:** 2009-11-19

**Authors:** Ignacio Marín

**Affiliations:** 1 Instituto de Biomedicina de Valencia, Consejo Superior de Investigaciones Científicas (IBV-CSIC), Valencia, Spain

## Abstract

**Background:**

Cullins are proteins involved in ubiquitination through their participation in multisubunit ubiquitin ligase complexes. In this study, I use comparative genomic data to establish the pattern of emergence and diversification of cullins in eukaryotes.

**Results:**

The available data indicate that there were three cullin genes before the unikont/bikont split, which I have called *Culα*, *Culβ *and *Culγ*. Fungal species have quite strictly conserved these three ancestral genes, with only occasional lineage-specific duplications. On the contrary, several additional genes appeared in the animal or plant lineages. For example, the human genes *Cul1*, *Cul2*, *Cul5*, *Cul7 *and *Parc *all derive from the ancestral *Culα *gene. These results, together with the available functional data, suggest that three different types of ubiquitin ligase cullin-containing complexes were already present in early eukaryotic evolution: 1) SCF-like complexes with Culα proteins; 2) Culβ/BTB complexes; and, 3) Complexes containing Culγ and DDB1-like proteins. Complexes containing elongins have arisen more recently and perhaps twice independently in animals and fungi.

**Conclusion:**

Most of the known types of cullin-containing ubiquitin ligase complexes are ancient. The available data suggest that, since the origin of eukaryotes, complex diversity has been mostly generated by combining closely related subunits, while radical innovations, giving rise to novel types of complexes, have been scarce. However, several protist groups not examined so far contain highly divergent cullins, indicating that additional types of complexes may exist.

## Background

Ubiquitination is a critical process in all eukaryotic organisms. It is involved in several essential functions, from the regulation of protein levels to roles in cellular signaling, DNA repair, endocytosis or gene expression regulation [1-4]. Ubiquitin ligases (E3s) are basic components of the ubiquitination system. They are a numerous and highly diverse group of enzymes able to transfer ubiquitin to the target proteins [1]. It has been observed that many E3s are single proteins. However, in other cases the ubiquitin ligase function is performed by multiprotein complexes. Particularly significant are cullin-RING ubiquitin ligases (CRLs), a diverse group of E3 complexes characterized by containing both a cullin family protein and a RING finger-containing protein. The roles of the cullin protein in this type of complex are quite well understood. Structural data indicate that cullins act as backbones that facilitate ubiquitination by correctly positioning both the RING finger-containing protein, that recruits the ubiquitin-conjugating enzyme (E2), and another protein present in the CRL complex, the substrate receptor, which confers substrate specificity. The CRL complexes also often contain one or more adaptor proteins, which at the same time bind the cullin and recruit the substrate receptor [5,6].

CRLs are the most abundant E3s. Their diversity is due to the fact that many alternative complexes can be generated in a combinatorial way: multiple related proteins may substitute each other to form similar but functionally distinct complexes. The number of CRLs may be very large: in all eukaryotic species analyzed in detail so far there are several cullins, related RING finger-containing proteins, several adaptors and, most especially, many alternative substrate receptors (e. g. potentially, there may be several hundreds in mammals) that participate in CRLs. Fortunately, and in spite of this extensive variability, analyses in multiple species has allowed to classify all complexes known so far into a few main CRL classes: 1) the Cullin/RING/Skp/F-box CRLs (historically known as SCF complexes, and to which I will refer generically as F-box CRLs) that contain proteins with F boxes as substrate receptors and Skp1 or related proteins as adaptors; 2) the Cullin/RING/BTB CRLs (BTB CRLs), characterized by lacking additional adaptors and containing proteins with BTB domains as substrate receptors, directly bound to the cullins; 3) the Cullin/RING/DDB/DCAFs CRLs (DDB CRLs) that contain proteins related to mammalian DDB1 as adaptors and often proteins with WD40 domains as substrate receptors; and, 4) Cullin/RING/Elongins/BC-box CRLs (BC-box CRLs), which contain one or two elongin proteins as adaptors and BC-box/SOCS-box containing proteins as substrate receptors (see reviews [5-9]).

It is known that CRLs regulate multiple cellular and developmental pathways in animals, fungi and plants, and certainly that may hold true for all free living eukaryotes (see e. g. refs. [5,6]). In addition of the intrinsic importance of cullins as critical players in ubiquitination control, they have recently received additional attention due to implication of mutations in cullin-encoding genes in several human diseases [10-13]. It is thus surprising that large-scale studies of the CRL complexes from a comparative point of view have not been hitherto performed. For example, no systematic efforts to determine the evolution of cullin proteins in the eukaryotes as a whole have been attempted. There were only some studies in which a few sequences were examined from an evolutionary point of view [14-18]. This has caused significant problems. A typical one is the assignation of the same names to genes in different species just because the proteins that they encode belong to complexes with similar units (for example, similar adaptors and substrate receptors), without any data actually supporting that those genes are orthologous. In addition, in some cases genes were named identically while being described independently in different species -- typically the names included the term cullin plus a consecutive number -- without considering at all their relationships. Thus, the current literature contains a significant degree of uncertainty about the similarities and differences of cullins and CRL complexes in different species, which may lead to inappropriate translations of the functional results obtained in one species to the rest. As a step to characterize the evolution of CRL complexes, I describe here the first comprehensive analysis of the evolution of cullin proteins. The results obtained in this study confirm several well-established ideas in the field, but also open some novel perspectives.

## Methods

### Generation of a database of cullin proteins

The protein sequences corresponding to the most conserved region of twelve cullin proteins were used in TblastN searches to find all members of this family present at the nr, est, gss, htgs or wgs databases of the National Center for Biotechnology Information (NCBI; http://www.ncbi.nlm.nih.gov/). These sequences were selected both to cover all the variation detected in previous analyses of cullin proteins and also to specifically check for all members of potential new families, most especially in protozoans. The selected genes derived from *Homo sapiens *(Cul3 and Cul7 proteins), *Drosophila melanogaster *(CG11261), *Caenorhabditis elegans *(Cul4), *Saccharomyces cerevisiae *(Cul8), *Debaryomyces hansenii *(Accession number CR382135.2), *Cyanidioschyzon merolae *(Acc. No. AP006495.1), *Plasmodium falciparum *(Acc. No. XM_961187.1), *Trypanosoma brucei *(Acc. Nos. XM_842334.1, XM_839532.1 and XM_838630.1) and *Leishmania major *(Acc. No. XM_001684442.1). The conserved region of the proteins used in these searches was homologous to amino acids 420 - 776 in human Cullin1. Once excluded partial sequences (< 300 amino acids), duplicates or nearly identical sequences (≥ 99% identity) and highly divergent sequences that could not be reliably aligned along the whole length of the selected region, I generated a database containing 490 sequences (available as Additional File 1). The cullin domain-containing APC2 proteins will not be considered here, given that their similarity with canonical cullins was too low. The rest of cullins, including the highly divergent cullin domain-containing CUL7 and PARC proteins of vertebrates, which were indeed easily detected and aligned, were all included in this study.

### Phylogenetic and structural analyses

Protein sequences were aligned using ClustalX 2.07 [19] and manually corrected using GeneDoc [20]. Multiple dendrograms (see Results) were then built using data extracted from that primary alignment. Three different procedures to generate those dendrograms were used, namely Neighbor joining (NJ), Maximum parsimony (MP) and Maximum likelihood (ML). The NJ tree was obtained using the routine in MEGA 4 [21] MP analyses were performed using PAUP* beta 10 version [22] and ML reconstructions were established using PhyML 2.4.4 [23]. For NJ, sites with gaps were treated with the pairwise deletion option (as recommended by [24]) and Kimura's correction implemented. Parameters for MP were as follows: 1) all sites included, gaps treated as unknown characters; 2) randomly generated trees used as seeds; 3) maximum number of trees saved equal to 100; and, 4) heuristic search using the subtree pruning-regrafting algorithm. Finally, for ML analyses, the BioNJ tree was used to start the iterative searches and the Blosum62 matrix was chosen to model amino acidic substitutions. Gaps are also treated by PhyML as unknown characters. Reliability of the topologies was tested in all cases by bootstrap analyses. 1000 bootstrap replicates were performed for the NJ and MP analyses and 200 for the ML analyses, which are much more computer intensive. Dendrograms were drawn using the tree editor of MEGA 4. Domains in cullin proteins were characterized using InterProScan [25].

## Results

### Characterization of the types of cullins present in animals, plants and fungi

The sequences of cullin proteins are very diverse, so general trees containing all the sequences found in the TblastN searches failed to unravel the relationships among the sequences of distant species (as an example, see the NJ tree for the 490 sequences in Additional File 2). In addition, structural analyses failed to detect significant features that might be used to establish relationships among proteins. InterProScan analyses showed that all cullins have a variable N-terminal end, which generally is detected as containing the InterPro domain IPR016159 ("Cullin repeat"), a central, highly conserved region that contains the InterPro domain IPR016158 ("Cullin homology domain") and a C terminus, also highly conserved, that includes the Pfam PF10557 domain ("Cullin_Nedd8 domain"), required for cullin neddylation, an essential step in CRL activity regulation (reviewed in refs. [6,8,9]). The two last domains were the ones included in the sequences that I examined. The only exceptions observed after sampling multiple representative sequences belonging to all the main groups of cullins detected in this study, were on one hand some *Plasmodium *proteins (e. g. *Plasmodium falciparum *Acc. No. NC_004327.1), which apparently lack the Cullin_Nedd8 domain and, on the other hand, the complex Cul7 and Parc proteins, which are encoded by genes derived from gene fusions, as we already described before [26,27].

**Figure 1:**
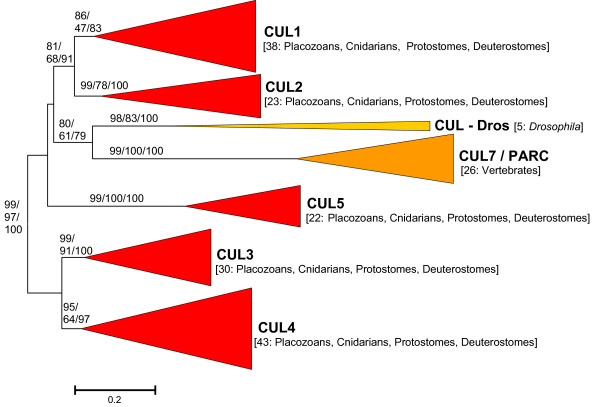
**Phylogenetic trees corresponding to animal cullin sequences**. The figure corresponds to the NJ tree, but the MP and ML results were topologically so similar that they are also included here. Numbers in the branches refer to bootstrap support, in percentages (order: NJ/MP/ML). Numbers in brackets refer to the number of sequences within each group. Five genes (red) have been found in all animal groups. Two other (orange) are vertebrate-specific or *Drosophila*-specific. Details of the sequences can be found in Additional File 3.

**Figure 2:**
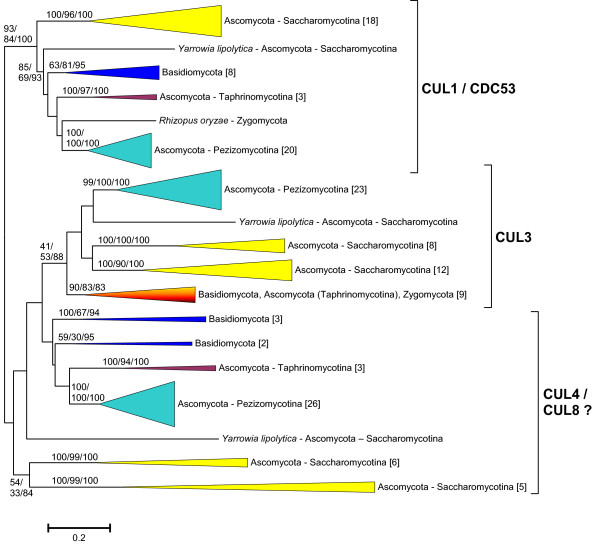
**Dendrogram showing the relationships among fungal cullins**. Bootstrap support and number of species in the groups are indicated as in Figure 1. Details of the sequences are described in Additional File 4.

**Figure 3:**
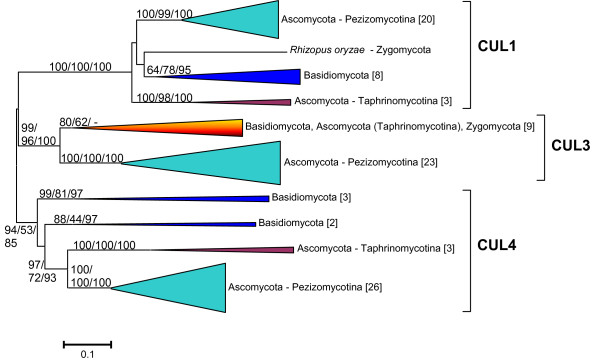
**Dendrogram showing the relationships among fungal cullins, after eliminating the saccharomycotina species**. Three highly suppported groups are apparent, which appeared before the ascomycota/basidiomycota split.

Given these difficulties, I decided to perform specific analyses which could be used as a starting point for a more general examination of the data. Figures 1, 2 and 3 shows a compact view of the phylogenetic trees obtained for sets corresponding to 187 animal sequences (Figure 1), 150 fungal sequences (Figure 2) and 128 sequences from bikonts (plants: 57 sequences; green and red algae: 11 sequences; stramenopiles: 17 sequences; alveolates: 19 sequences; excavates: 24 sequences; Figure 3). The details of the trees, including the accession numbers of the sequences, can be found as Additional Files 3, 4 and 5. Additional file 6 contains the sequences included in the animal, fungal and bikont analyses as separate datasets.

Results in Figure 1 are very well supported and confirm the accepted classification of animal cullins into six main orthology groups, CUL1, CUL2, CUL3; CUL4, CUL5 and CUL7/PARC which respectively include the human genes *Cul1*, *Cul2*, *Cul3*, *Cul4A *and *Cul4B *(both in the CUL4 group), *Cul5, Cul7 *and *Parc *(the last two in the CUL7/PARC group). For five of those groups (CUL1 - CUL5), I found genes in the placozoan *Trichoplax adhaerens *and the cnidarian *Nematostella vectensis*, indicating that they originated before the split of the different animal lineages. On the contrary, genes in the CUL7/PARC group are restricted to chordates and some species of the *Drosophila *genus contain an additional gene that cannot be ascribed to any of the main classes (forming the CUL-Dros group in Figure 1). We can assume this is a recent fly-specific duplicate that diverged extensively from the rest of cullins in a short period of time.

To interpret the evolutionary history of fungal cullins is more difficult. Figure 2 shows the main groups detected for which there is bootstrap support. It is very significant that, except for some species-specific duplicates, all fungi have three cullin genes. Thus, the simplest hypothesis to explain the results shown in Figure 2 is that three genes existed before the ascomycetes/basidiomycetes split. These three genes would correspond respectively to the *CDC53*, *CUL3 *and *CUL8/RTT101 *genes of *Saccharomyces cerevisiae *or, also respectively, to the *cul1*, *cul3 *and *cul4 *genes of *Schizosaccharomyces pombe*. The problem with this hypothesis is the absence of a strong support for the putative Cul3 and Cul4/Cul8 branches (see Figure 2). Especially troublesome is that the *CUL8/RTT101 *genes of saccharomycotina species are very different from the *cul4-like *genes of the rest of ascomycetes and basidiomycetes (see also Figure 2). This can be explained in two different ways. One option is that *CUL8/RTT101 *genes in saccharomycotina are indeed *cul4-like *genes that have suffered an acceleration of their evolutionary rates which makes difficult to determine their precise phylogenetic position. A second, albeit less parsimonious option, is that saccharomycotina species have lost their ancestral *cul4-like *gene and in parallel an additional cullin gene arose by duplication, giving rise to the *CUL8/RTT101 *gene. In any case, no matter which of those two possible explanations is true, it should be possible to determine with precision the phylogenetic relationships of all the rest of fungal cullin genes, once saccharomycotina species are eliminated. This is shown in Figure 3, in which it is clear that the cullins in the rest of fungi all belong to one of the three groups, Cul1, Cul3 or Cul4. The conclusion is that three genes existed when fungi emerged.

**Figure 4:**
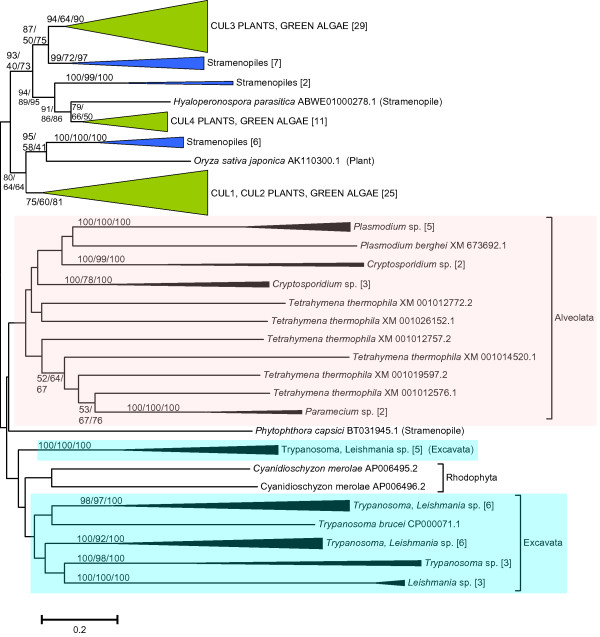
**Dendrogram of cullin sequences obtained from bikont species**. Notice the three groups in plants and stramenopiles and the multiple highly divergent groups in alveolata (pink) and excavata (blue). Bootstrap support and number of species per branch are indicated as in the previous figures (again bootstrap order: NJ/MP/ML). Details can be found in Additional File 5.

Figure 4 shows the result for the set of cullin sequences obtained from bikont species. Interestingly, there are three main groups, highly supported by bootstrap analyses, which include sequences from viridiplantae (plants, green algae) and stramenopiles. Multiple, very similar paralogous genes, evidently associated to their well-known genome duplications, appear in most plant species (see details in Additional file 5). For example, one of the three main groups contain three *Arabidopsis thaliana *genes (known as *Cul1*, *Cul2 *and *Cul2-like/Cul2b*, this last one a likely pseudogen), a second group includes two genes (named *Cul3a *and *Cul3b*) and the third just a single gene, *Cul4*. This agrees with previous results ([16-18]; additionally, these authors described small cullin-like proteins which did not align along the whole length of the region that I considered in this study and therefore were discarded). The fact that in plants, stramenopiles and fungi the basic, ancestral number of cullin genes is three suggests that these genes may, in origin, be the same. This possibility will be explored in the next section.

In addition to the three main groups present in plants and stramenopiles, several highly divergent sequences are detected in other species, most of them belonging to the alveolata and excavata. Alveolata species have from 1 (in a particular *Plasmodium *species) to 6 genes (as in *Tetrahymena thermophila*). When duplicates are present, they are all very similar, implying recent duplication events in particular lineages (see details in Additional File 5). The genes of excavata species, on the other hand, appear as four - five very distinct groups (Figure 4). No obvious relationships of these highly divergent genes with the plant or stramenopile cullin genes could be traced.

### Reconciling the phylogenetic trees of animals, fungi and plants

The results shown in the previous section confirm or clarify several relevant aspects of the origin and evolution of cullins, but on the other hand open new significant questions. Of particular interest is to correlate the known biochemical roles of the different cullins (e. g. in which type of CRL they participate) with their diversification. A related point is to establish when the different types of cullins emerged. Data presented in the previous section are compatible with the presence of at least three cullin genes before the unikont/bikont split, followed by lineage-specific duplications. However, more complex alternatives, with some genes disappearing and others emerging in multiple lineages can also be put forward to explain those results. Therefore, I decided to further explore the data in order to determine the most likely scenario for the early evolution of cullins.

**Figure 5:**
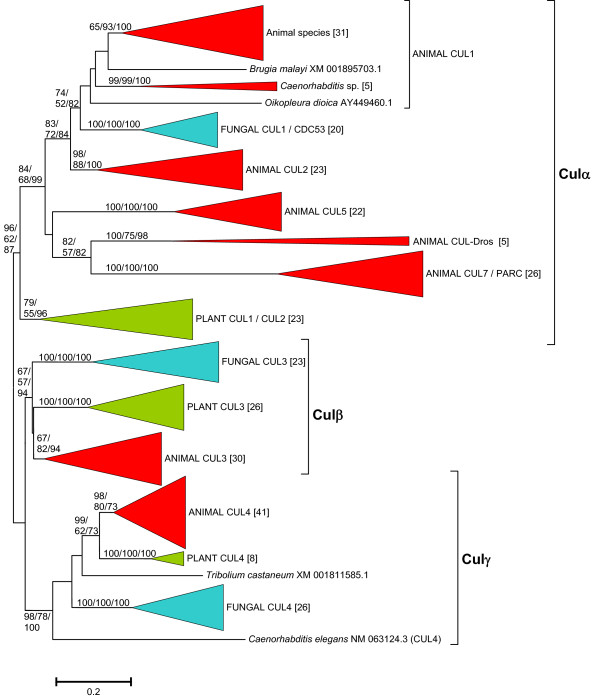
**Phylogenetic relationships obtained for all animal and plant sequences plus sequences from pezizomycotina fungi**. The three groups, corresponding to the ancestral *Culα*, *Culβ *and *Culγ *genes, are highly supported by the alternative methods of phylogenetic reconstruction (NJ/MP/ML). Details in Additional File 6.

Given that I found, as described in the previous section, that the cullins of both some fungal groups and some bikont groups are highly divergent, I decided to exclude those problematic sequences and focus the analyses on a more limited dataset. Particularly, in Figures 2 and 3, I showed that the sequences of the cullins of a type of ascomycetes, the pezizomycotina, form three compact groups that would correspond to the fungal *Cul1*, *Cul3 *and *Cul4 *genes already discussed above. Therefore, I decided to use these slowly-evolving sequences from pezizomycotina species as representatives of fungal cullins in more comprehensive analyses. Figure 5 shows the results for the analyses that include all available animal and plant sequences plus those pezizomycotina-derived sequences (details can be found in Additional File 7). The question that the results shown in Figure 5 try to answer is whether, as suggested by the previous analyses, just three genes existed before the unikont/bikont split. As indicated in that figure, the results obtained are totally compatible with that possibility. The deepest dichotomy shown in Figure 5 separates a group formed by several animal cullin genes (*Cul1*, *Cul2*, *Cul5*, *Cul-Dros*, *Cul7/Parc*) a single fungal cullin gene (*Cul1*, also known as *Cdc53*) and two recently duplicated plant cullin genes (*Cul1 *and *Cul2*) from two other groups which respectively include the cullin genes so far named *Cul3 *and *Cul4 *in animals, plants and fungi. As indicated in Figure 5, the three groups may have emerged from the diversification of single ancestral cullin genes, all of them originated very early in eukaryotic evolution, before the separation of unikonts and bikonts. I have named these ancestral genes, and the groups of genes that derive from them, as *Culα*, *Culβ *and *Culγ *(Figure 5).

Table 1 shows the functional data that also support the early emergence of three cullin genes. As shown in that table, genes included in the Culα group in Figure 4 encode proteins that are very often part of F-box CRLs [28-34], while *Culβ *genes in general encode cullins that interact with BTB domain-containing proteins [35-40] and the products of *Culγ *genes are included in DDB CRL complexes [41-50]. There are however exceptions to this pattern. The best established is that several animal *Culα *genes (*Cul2*, *Cul5*) are known to interact with substrate receptors different from F-box proteins (Table 1; [51-55]). In addition, there is also a work suggesting that *S. cerevisiae *Cul3 protein may interact with BC-box-containing elongins instead of BTB proteins [56]. All these exceptions may be easily interpreted as secondary lineage- and protein-specific diversifications. On one hand, the available data indicate that the emergence of multiple *Culα *genes in animals (Figure 1) has been accompanied by a diversification of the partners of the proteins they encode (Table 1). On the other hand, if indeed the Cul3/BC-box interaction exists, the results for *S. cerevisiae *Cul3 might be interpreted as a recent, drastic modification of its ancestral function. However, this is unlikely. It would mean that *Saccharomyces *does not have any cullin-BTB complex, but this type of complex has been found in all other organisms for which there is functional data. An alternative would be that Cul3 proteins in *S. cerevisiae *may be forming part of two different types of CRLs, one in which the adaptor is a BTB protein and a second one in which the adaptors are elongins. This second option predicts that a Cul3-BTB complex should be found in *Saccharomyces*. Table 1 also supports the idea that *S. cerevisiae CUL8/RTT101 *is indeed, and in spite of the low sequence similarity shown in Figure 2, a true ortholog of the *Cul4 *genes in other fungi, given its interaction with a DDB-like protein [49].

**Table 1:** Types of cullin complexes characterized so far in different organisms.

	Culα	Culβ	Culγ
**ANIMALS**	**Cul1 - Skp1 - F-box proteins **[31,32] **Cul7 - Skp1 - F-box proteins **[34] Cul2 -Elongins B, C - BC box proteins [51,52] Cul5 - Elongins B, C - BC/SOCS box proteins [53-55]	**Cul3 - BTB **[35]	**Cul4a, Cul4b - DDB1 - DCAFs **[41,44-48,50]

**FUNGI** ***(S. cerevisiae)***	**Cdc53 - Skp - F-box proteins **[28,29]	Cul3 - Elongin C - Elongin A (BC box) ?? [56]	**Cul8/Rtt101-Mms1 (DDB-like) **[49]

**FUNGI** ***(S. pombe)***	**Cul1 - Skp - F-box proteins **[30]	**Cul3 - BTB (*S. pombe*) **[36]	**Cul4 - Rik 1 (DDB-like) **[42]

**PLANTS**	**Cul1 - Ask (Skp1-like) - F-box **[33]	**Cul3a, Cul3b - BTB **[37-40]	**Cul4 - DDB1 - DET1 **[43,44]

***Ancestral complexes***	**Culα- Skp1 - F-box CRL**	**Culβ- BTB CRL**	**Culγ - DDB1 CRL**

References are indicated in brackets. The respective cullins, adaptors (if present) and substrate receptors are indicated. The ancestral complexes were deduced by considering the phylogenetic range of each association. In bold, data that coincide with the ancestral situation. Most animal data were obtained from mammalian species, although some have been also characterized in invertebrates. Fungal data come from the model species *Saccharomyces cerevisiae *and *Schizosaccharomyces pombe*, as indicated. Plant data derive from *Arabidopsis thaliana*. The question marks refer to the fact that the interaction Cul3/elongins in *S. cerevisiae *remains unconfirmed.

## Discussion

The results obtained are compatible with the presence of three cullin genes (which I have named *Culα*, *Culβ *and *Culγ*) in early eukaryotic evolution. This hypothesis is supported by the independent results in the different groups on which I have focused this study (mainly animals, fungi and plants; Figures 1, 2, 3 and 4), and especially by the combined results when all the sequences in animals and plants and a selected group of slowly evolving sequences from fungi are analyzed together (Figure 5) and fit well with the available functional data (Table 1). We can thus conclude that, in spite of a substantial sequence divergence that complicates the analyses, orthology relationships can be established among cullins of distantly related eukaryotes. In general, the associations found agree well with previous results. For example, the close relationships among *Cul3 *and *Cul4 *genes in animals, fungi and plants were observed before [17,18]. However, my results also provide some additional interesting information which contributes to understand the relationships among all cullins. For example, it can be deduced from the results shown in Figures 1 and 5 that the animal genes *Cul1*, *Cul2*, *Cul5*, *Cul7*, *Parc *and *Cul-Dros *all derive from the ancestral *Culα *gene. These means that they are all equally related to the *Cul1*/*Cdc53 *genes in yeasts and to the *Cul1/Cul2 *genes in plants, a result that is very significant if we want to compare functional results in different model species. These results also demonstrate that it is inappropriate to use the name *Cul2 *for plant genes which in fact are plant-specific duplicates, totally unrelated to animal *Cul2 *genes. In fact, previous analyses had failed to properly situate the plant Cul1/Cul2 branch [17,18], which I have shown here to clearly correspond to the Culα plant sequences (Figure 4). A final, logical conclusion of the results presented here is that to generate a revised cullin nomenclature based on evolutionary relationships would be advisable. A logical step would be to include in the name of the genes an indication to which group (α, β or γ) they belong.

Once established the most likely orthology relationships among genes, it is possible to evaluate when each type of CRL complex may have arisen. We may deduce in which type of complexes the three different ancestral cullins were involved, by considering what is currently known in model species (Table 1). The most parsimonious conclusion is that each type of cullin was already involved in a different type of complex. Therefore, it can be hypothesized that there were three types of cullin complexes in early eukaryotes: F-box CRLs, BTB CRLs and DDB CRLs (see data in Table 1). The rest of complexes must have arisen more recently. Thus, both the animal Cul2/Elongin and Cul5/Elongin complexes (Table 1) must be animal-specific, considering the relatively recent emergence of those two genes, already described. Finally, the description of a Cul3/Elongin complex in the yeast *Saccharomyces cerevisiae *(Table 1) is incongruent. The fact that no other Cul3 protein has ever been found to interact with elongins and that the elongin-containing complexes in animals only involve animal-specific cullins, as I just mentioned, suggest that all complexes that include adaptors with BC boxes (VHL, SOCS proteins, Elongin A, etc.; see details in the references listed in Table 1) emerged relatively recently. The presence of those complexes in both animals and (if confirmed) in *Saccharomyces *must therefore be due to parallel evolution: the same novel interaction between cullins and BC box-containing proteins emerged twice independently. This conclusion would be falsified only if additional Cul3/Elongin A complexes in animals or plants are found. On the other hand, so far no Cul3/BTB CRL complex has been described in *Saccharomyces*. However, the finding of such complexes in plants, animals and even other fungi strongly suggest that they must exist also in budding yeast species. Perhaps, as I already suggested above, this means that Cul3 in *Saccharomyces *participates in two different complexes, one of them involving elongins and a second one involving BTB-containing adaptors.

A final consideration is that the discovery of multiple, highly divergent multiple cullin genes in some protozoans, and especially species of the Alveolata and Excavata groups (Figure 3) suggests that the spectrum of possible CRL complexes in eukaryotes may be much wider than the scientific community has so far established. At a more local level, the substantial diversification of the Cul4 sequences in some fungi, especially the saccharomycotina (Figure 2), may also be an indication of them having acquired peculiar functional features. These possibilities may be experimentally explored in the near future. Finally, it is also interesting to point out that APC2 genes, not considered here given its low degree of similarity, may all derive from a fourth gene with a cullin domain that also emerged before the unikont/bikont split [57-60].

## Conclusion

The origin of most of the known types of CRLs is ancient. The current diversity of CRL complexes in animals, plants and fungi is mostly explained by the emergence of different combinations of related proteins to give rise to multiple similar complexes. So far, a single type of complex is known that emerged since the unikont/bikont split, and perhaps twice independently in animals and fungi. However, the characterization of CRL complexes in additional protists (e. g. alveolata, excavata) may lead to the discovery of additional novel types of complexes of recent origin.

## Supplementary Material

Additional file 1 **Cullin sequences used in this work**. 490 cullin sequences in Fasta format. Click here for file

Additional file 2 **NJ tree for all cullin sequences**. contains a NJ-based phylogenetic tree for the whole dataset (490 sequences). The groups indicated were deduced at the end of this work. Notice the low bootstrap support for most branches. Click here for file

Additional file 3 **NJ tree for animal cullins**. data for Figure 1 of the paper (187 animal sequences). Click here for file

Additional file 4 **NJ tree for fungal cullins**. data for Figures 2 and 3 of the paper (150 fungal sequences). Click here for file

Additional file 5 **NJ tree for bikont cullins**. data for Figure 4 of the paper (128 bikont sequences). Click here for file

Additional file 6 **Cullin sequences of animals, fungi and bikont species**. sequences corresponding to the analyses shown in Figure 1 (187 animal sequences), Figure 2 (150 fungal sequences) and Figure 4 (128 bikont sequences). Click here for file

Additional file 7 **NJ tree for animal, plant and pezizomycotina sequences**. data for Figure 5 of the paper (313 sequences). (This can be opened using MEGA 4 [http://www.megasoftware.net/]). Click here for file
