# Supplementary material for: Diversification of the cullin family
Source: BMC Evol Biol. 2009 Nov 19;9:267. doi: 10.1186/1471-2148-9-267 (PMC2785787; doi:10.1186/1471-2148-9-267)
Supplement: Supplementary file 1 [file 1471-2148-9-267-S2.DOC]

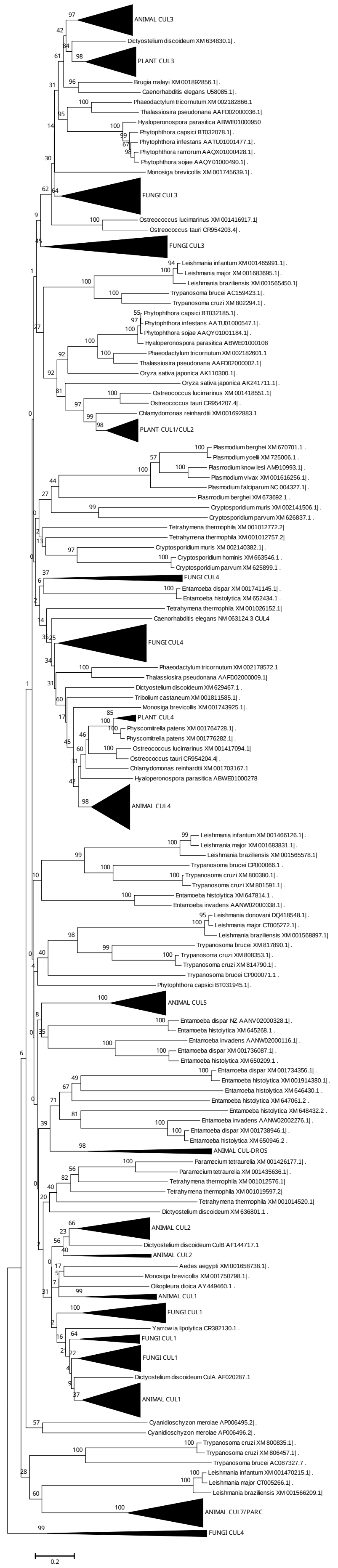
ANIMAL CUL3
Dictyostelium discoideum XM 634830.1| .
PLANT CUL3
Brugia malayi XM 001892856.1| .
Caenorhabditis elegans U58085.1| .
Phaeodactylum tricornutum XM 002182866.1
Thalassiosira pseudonana AAFD02000036.1|
Hyaloperonospora parasitica ABWE01000950
Phytophthora capsici BT032078.1| .
Phytophthora infestans AATU01001477.1| .
Phytophthora ramorum AAQX01000428.1| .
Phytophthora sojae AAQY01000490.1| .
Monosiga brevicollis XM 001745639.1| .
FUNGI CUL3
Ostreococcus lucimarinus XM 001416917.1|
Ostreococcus tauri CR954203.4| .
FUNGI CUL3
Leishmania infantum XM 001465991.1| .
Leishmania major XM 001683695.1| .
Leishmania braziliensis XM 001565450.1|
Trypanosoma brucei AC159423.1| .
Trypanosoma cruzi XM 802294.1| .
Phytophthora capsici BT032185.1| .
Phytophthora infestans AATU01000547.1| .
Phytophthora sojae AAQY01001184.1| .
Hyaloperonospora parasitica ABWE01000108
Phaeodactylum tricornutum XM 002182601.1
Thalassiosira pseudonana AAFD02000002.1|
Oryza sativa japonica AK110300.1| .
Oryza sativa japonica AK241711.1| .
Ostreococcus lucimarinus XM 001418551.1|
Ostreococcus tauri CR954207.4| .
Chlamydomonas reinhardtii XM 001692883.1
PLANT CUL1/CUL2
Plasmodium berghei XM 670701.1 .
Plasmodium yoelii XM 725006.1 .
Plasmodium know lesi AM910993.1| .
Plasmodium vivax XM 001616256.1| .
Plasmodium falciparum NC 004327.1| .
Plasmodium berghei XM 673692.1 .
Cryptosporidium muris XM 002141506.1| .
Cryptosporidium parvum XM 626837.1 .
Tetrahymena thermophila XM 001012772.2|
Tetrahymena thermophila XM 001012757.2|
Cryptosporidium muris XM 002140382.1| .
Cryptosporidium hominis XM 663546.1 .
Cryptosporidium parvum XM 625899.1 .
FUNGI CUL4
Entamoeba dispar XM 001741145.1| .
Entamoeba histolytica XM 652434.1 .
Tetrahymena thermophila XM 001026152.1|
Caenorhabditis elegans NM 063124.3 CUL4
FUNGI CUL4
Phaeodactylum tricornutum XM 002178572.1
Thalassiosira pseudonana AAFD02000009.1|
Dictyostelium discoideum XM 629467.1 .
Tribolium castaneum XM 001811585.1| .
Monosiga brevicollis XM 001743925.1| .
PLANT CUL4
Physcomitrella patens XM 001764728.1| .
Physcomitrella patens XM 001776282.1| .
Ostreococcus lucimarinus XM 001417094.1|
Ostreococcus tauri CR954204.4| .
Chlamydomonas reinhardtii XM 001703167.1
Hyaloperonospora parasitica ABWE01000278
ANIMAL CUL4
Leishmania infantum XM 001466126.1| .
Leishmania major XM 001683831.1| .
Leishmania braziliensis XM 001565578.1|
Trypanosoma brucei CP000066.1 .
Trypanosoma cruzi XM 800380.1| .
Trypanosoma cruzi XM 801591.1| .
Entamoeba histolytica XM 647814.1 .
Entamoeba invadens AANW02000338.1| .
Leishmania donovani DQ418548.1| .
Leishmania major CT005272.1| .
Leishmania braziliensis XM 001568897.1|
Trypanosoma brucei XM 817890.1| .
Trypanosoma cruzi XM 808353.1| .
Trypanosoma cruzi XM 814790.1| .
Trypanosoma brucei CP000071.1 .
Phytophthora capsici BT031945.1| .
ANIMAL CUL5
Entamoeba dispar NZ AANV02000328.1| .
Entamoeba histolytica XM 645268.1 .
Entamoeba invadens AANW02000116.1| .
Entamoeba dispar XM 001736087.1| .
Entamoeba histolytica XM 650209.1 .
Entamoeba dispar XM 001734356.1| .
Entamoeba histolytica XM 001914380.1| .
Entamoeba histolytica XM 646430.1 .
Entamoeba histolytica XM 647061.2 .
Entamoeba histolytica XM 648432.2 .
Entamoeba invadens AANW02002276.1| .
Entamoeba dispar XM 001738946.1| .
Entamoeba histolytica XM 650946.2 .
ANIMAL CUL-DROS
Paramecium tetraurelia XM 001426177.1| .
Paramecium tetraurelia XM 001435636.1| .
Tetrahymena thermophila XM 001012576.1|
Tetrahymena thermophila XM 001019597.2|
Tetrahymena thermophila XM 001014520.1|
Dictyostelium discoideum XM 636801.1 .
ANIMAL CUL2
Dictyostelium discoideum CulB AF144717.1
ANIMAL CUL2
Aedes aegypti XM 001658738.1| .
Monosiga brevicollis XM 001750798.1| .
Oikopleura dioica AY449460.1 .
ANIMAL CUL1
FUNGI CUL1
Yarrow ia lipolytica CR382130.1 .
FUNGI CUL1
FUNGI CUL1
Dictyostelium discoideum CulA AF020287.1
ANIMAL CUL1
Cyanidioschyzon merolae AP006495.2| .
Cyanidioschyzon merolae AP006496.2| .
Trypanosoma cruzi XM 800835.1| .
Trypanosoma cruzi XM 806457.1| .
Trypanosoma brucei AC087327.7 .
Leishmania infantum XM 001470215.1| .
Leishmania major CT005266.1| .
Leishmania braziliensis XM 001566209.1|
ANIMAL CUL7/PARC
FUNGI CUL4
0.2
