# Supplementary material for: Diversification of the cullin family
Source: BMC Evol Biol. 2009 Nov 19;9:267. doi: 10.1186/1471-2148-9-267 (PMC2785787; doi:10.1186/1471-2148-9-267)
Supplement: Supplementary file 2 [file 1471-2148-9-267-S3.DOC]

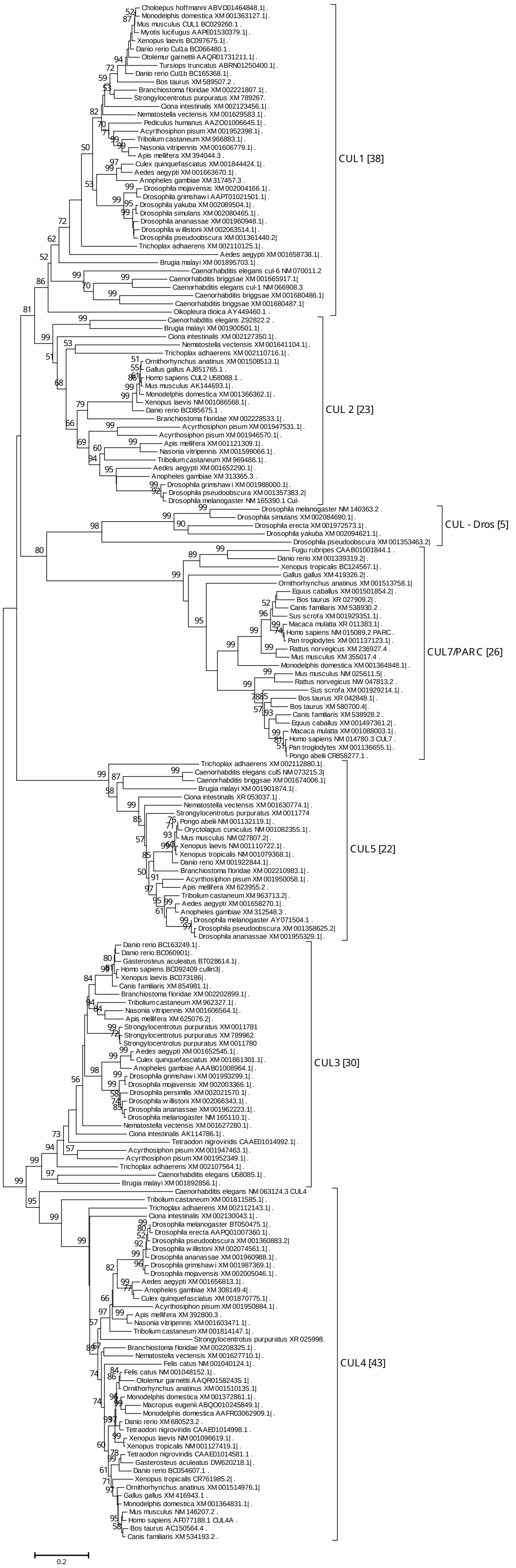

CUL1 [38]
CUL 2 [23]
CUL - Dros [5]
CUL7/PARC [26]
CUL5 [22]
CUL3 [30]
CUL4 [43]
0.2
