# Supplementary material for: Diversification of the cullin family
Source: BMC Evol Biol. 2009 Nov 19;9:267. doi: 10.1186/1471-2148-9-267 (PMC2785787; doi:10.1186/1471-2148-9-267)
Supplement: Supplementary file 3 [file 1471-2148-9-267-S4.DOC]

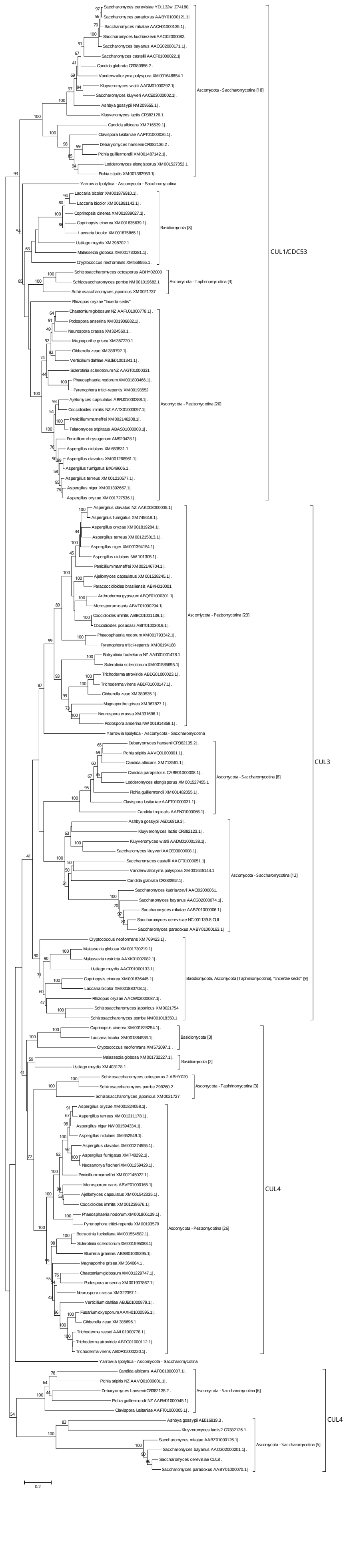

Ascomycota - Saccharomycotina [18]
Yarrowia lipolytica - Ascomycota - Saccharomycotina
Basidiomycota [8]
Ascomycota - Taphrinomycotina [3]
Rhizopus oryzae "Incerta sedis"
Ascomycota - Pezizomycotina [20]
CUL1/CDC53
Ascomycota - Pezizomycotina [23]
Yarrowia lipolytica - Ascomycota - Saccharomycotina
Ascomycota - Saccharomycotina [8]
Ascomycota - Saccharomycotina [12]
Basidiomycota, Ascomycota (Taphrinomycotina), "Incertae sedis" [9]
CUL3
Basidiomycota [3]
Basidiomycota [2]
Ascomycota - Taphrinomycotina [3]
Ascomycota - Pezizomycotina [26]
CUL4
Yarrowia lipolytica - Ascomycota - Saccharomycotina
Ascomycota - Saccharomycotina [6]
Ascomycota - Saccharomycotina [5]
CUL4
0.2
