# Supplementary material for: Diversification of the cullin family
Source: BMC Evol Biol. 2009 Nov 19;9:267. doi: 10.1186/1471-2148-9-267 (PMC2785787; doi:10.1186/1471-2148-9-267)
Supplement: Supplementary file 4 [file 1471-2148-9-267-S5.DOC]

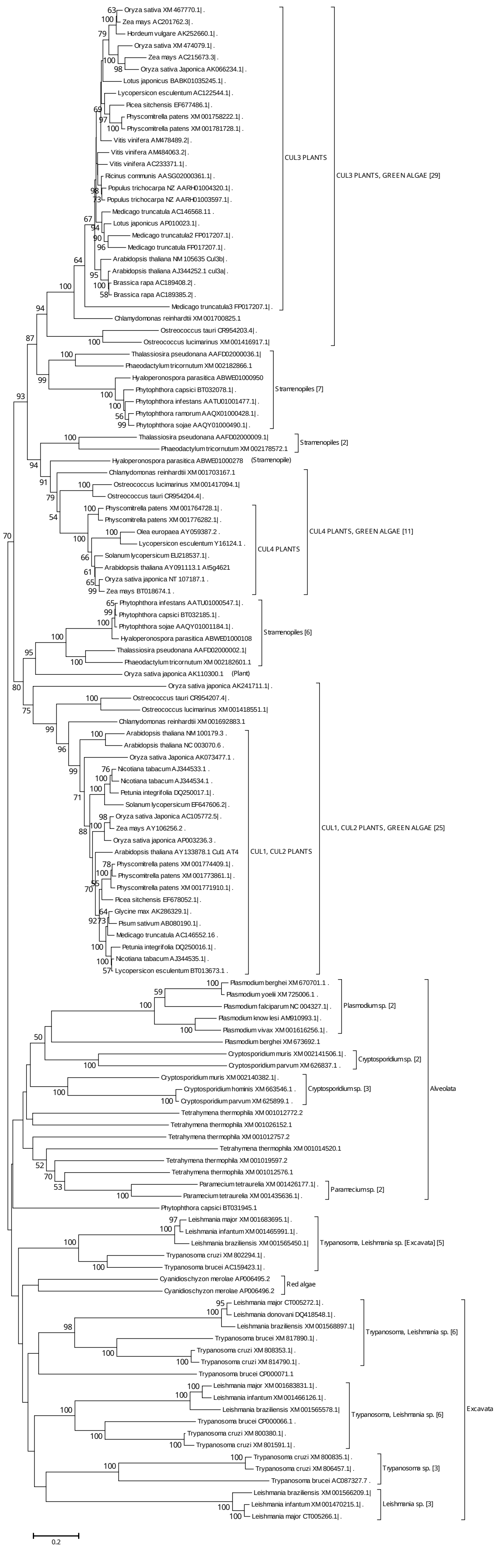

Oryza sativa XM 467770.1| .
Zea mays AC201762.3| .
Hordeum vulgare AK252660.1| .
Oryza sativa XM 474079.1| .
Zea mays AC215673.3| .
Oryza sativa Japonica AK066234.1| .
Lotus japonicus BABK01035245.1| .
Lycopersicon esculentum AC122544.1| .
Picea sitchensis EF677486.1| .
Physcomitrella patens XM 001758222.1| .
Physcomitrella patens XM 001781728.1| .
Vitis vinifera AM478489.2| .
Vitis vinifera AM484063.2| .
Vitis vinifera AC233371.1| .
Ricinus communis AASG02000361.1| .
Populus trichocarpa NZ AARH01004320.1| .
Populus trichocarpa NZ AARH01003597.1| .
Medicago truncatula AC146568.11 .
Lotus japonicus AP010023.1| .
Medicago truncatula2 FP017207.1| .
Medicago truncatula FP017207.1| .
Arabidopsis thaliana NM 105635 Cul3b| .
Arabidopsis thaliana AJ344252.1 cul3a| .
Brassica rapa AC189408.2| .
Brassica rapa AC189385.2| .
Medicago truncatula3 FP017207.1| .
Chlamydomonas reinhardtii XM 001700825.1
Ostreococcus tauri CR954203.4| .
Ostreococcus lucimarinus XM 001416917.1| .
CUL3 PLANTS
CUL3 PLANTS, GREEN ALGAE [29]
Thalassiosira pseudonana AAFD02000036.1|
Phaeodactylum tricornutum XM 002182866.1
Hyaloperonospora parasitica ABWE01000950
Phytophthora capsici BT032078.1| .
Phytophthora infestans AATU01001477.1| .
Phytophthora ramorum AAQX01000428.1| .
Phytophthora sojae AAQY01000490.1| .
Stramenopiles [7]
Thalassiosira pseudonana AAFD02000009.1|
Phaeodactylum tricornutum XM 002178572.1
Stramenopiles [2]
Hyaloperonospora parasitica ABWE01000278 (Stramenopile)
Chlamydomonas reinhardtii XM 001703167.1
Ostreococcus lucimarinus XM 001417094.1|
Ostreococcus tauri CR954204.4| .
Physcomitrella patens XM 001764728.1| .
Physcomitrella patens XM 001776282.1| .
Olea europaea AY059387.2 .
Lycopersicon esculentum Y16124.1 .
Solanum lycopersicum EU218537.1| .
Arabidopsis thaliana AY091113.1 At5g4621
Oryza sativa japonica NT 107187.1 .
Zea mays BT018674.1 .
CUL4 PLANTS
CUL4 PLANTS, GREEN ALGAE [11]
Phytophthora infestans AATU01000547.1| .
Phytophthora capsici BT032185.1| .
Phytophthora sojae AAQY01001184.1| .
Hyaloperonospora parasitica ABWE01000108
Thalassiosira pseudonana AAFD02000002.1|
Phaeodactylum tricornutum XM 002182601.1
Stramenopiles [6]
Oryza sativa japonica AK110300.1 (Plant)
Oryza sativa japonica AK241711.1| .
Ostreococcus tauri CR954207.4| .
Ostreococcus lucimarinus XM 001418551.1|
Chlamydomonas reinhardtii XM 001692883.1
Arabidopsis thaliana NM 100179.3 .
Arabidopsis thaliana NC 003070.6 .
Oryza sativa Japonica AK073477.1 .
Nicotiana tabacum AJ344533.1 .
Nicotiana tabacum AJ344534.1 .
Petunia integrifolia DQ250017.1| .
Solanum lycopersicum EF647606.2| .
Oryza sativa Japonica AC105772.5| .
Zea mays AY106256.2 .
Oryza sativa japonica AP003236.3 .
Arabidopsis thaliana AY133878.1 Cul1 AT4
Physcomitrella patens XM 001774409.1| .
Physcomitrella patens XM 001773861.1| .
Physcomitrella patens XM 001771910.1| .
Picea sitchensis EF678052.1| .
Glycine max AK286329.1| .
Pisum sativum AB080190.1| .
Medicago truncatula AC146552.16 .
Petunia integrifolia DQ250016.1| .
Nicotiana tabacum AJ344535.1| .
Lycopersicon esculentum BT013673.1 .
CUL1, CUL2 PLANTS
CUL1, CUL2 PLANTS, GREEN ALGAE [25]
Plasmodium berghei XM 670701.1 .
Plasmodium yoelii XM 725006.1 .
Plasmodium falciparum NC 004327.1| .
Plasmodium knowlesi AM910993.1| .
Plasmodium vivax XM 001616256.1| .
Plasmodium sp. [2]
Plasmodium berghei XM 673692.1
Cryptosporidium muris XM 002141506.1| .
Cryptosporidium parvum XM 626837.1 .
Cryptosporidium sp. [2]
Cryptosporidium muris XM 002140382.1| .
Cryptosporidium hominis XM 663546.1 .
Cryptosporidium parvum XM 625899.1 .
Cryptosporidium sp. [3]
Tetrahymena thermophila XM 001012772.2
Tetrahymena thermophila XM 001026152.1
Tetrahymena thermophila XM 001012757.2
Tetrahymena thermophila XM 001014520.1
Tetrahymena thermophila XM 001019597.2
Tetrahymena thermophila XM 001012576.1
Paramecium tetraurelia XM 001426177.1| .
Paramecium tetraurelia XM 001435636.1| .
Paramecium sp. [2]
Alveolata
Phytophthora capsici BT031945.1
Leishmania major XM 001683695.1| .
Leishmania infantum XM 001465991.1| .
Leishmania braziliensis XM 001565450.1|
Trypanosoma cruzi XM 802294.1| .
Trypanosoma brucei AC159423.1| .
Trypanosoma, Leishmania sp. [Excavata] [5]
Cyanidioschyzon merolae AP006495.2
Cyanidioschyzon merolae AP006496.2
Red algae
Leishmania major CT005272.1| .
Leishmania donovani DQ418548.1| .
Leishmania braziliensis XM 001568897.1|
Trypanosoma brucei XM 817890.1| .
Trypanosoma cruzi XM 808353.1| .
Trypanosoma cruzi XM 814790.1| .
Trypanosoma, Leishmania sp. [6]
Trypanosoma brucei CP000071.1
Leishmania major XM 001683831.1| .
Leishmania infantum XM 001466126.1| .
Leishmania braziliensis XM 001565578.1|
Trypanosoma brucei CP000066.1 .
Trypanosoma cruzi XM 800380.1| .
Trypanosoma cruzi XM 801591.1| .
Trypanosoma, Leishmania sp. [6]
Trypanosoma cruzi XM 800835.1| .
Trypanosoma cruzi XM 806457.1| .
Trypanosoma brucei AC087327.7 .
Trypanosoma sp. [3]
Leishmania braziliensis XM 001566209.1|
Leishmania infantum XM 001470215.1| .
Leishmania major CT005266.1| .
Leishmania sp. [3]
Excavata
0.2
